# In silico study of medical decision-making for rare diseases: heterogeneity of decision-makers in a population improves overall benefit

**DOI:** 10.7717/peerj.5677

**Published:** 2018-09-25

**Authors:** Juan Wang, Ryo Yamada

**Affiliations:** Unit of Statistical Genetics, Center for Genomic Medicine, Graduate School of Medicine, Kyoto University, Kyoto, Japan

**Keywords:** Heterogeneity, Rare disease, Decision-making, Self-decision

## Abstract

**Background:**

Medical decision-making is difficult when information is limited due to its rareness. For example, there are two treatment options for patients affected by a rare disease with high lethality. The information about both treatment effects is unavailable or very limited. Patients are inclined to accept one of the interventions rather than waiting for death, but they are reluctant to be assigned the inferior one. While a single patient selects one treatment that seems better based on the limited information, he or she loses the chance to select the other treatment, which may be the better option. This is the so-called dilemma between exploitation (enjoying the benefits of using current knowledge) and exploration (taking the risk to obtain new knowledge). In clinical settings, the statistical advice for individual patients seems to be the maximum expected success rate or something equivalent and patients’ selections tend to be homogeneous, which does not solve the dilemma. In this study, our aim is to investigate the effects of the heterogeneity of decision-makers in the decision process.

**Methods:**

Here, we proposed a decision strategy that introduced the heterogeneity of decision-makers by considering patients’ self-decisions where the patients’ heterogeneous attitudes towards the treatment are integrated into the probabilistic utility function based on the Beta Bayesian posterior. Based on the context of two-armed bandit treatment options with limited information, we compared the overall success rate of treatment between our heterogeneous decision strategy and a homogeneous decision strategy that is defined to select the treatment with the largest posterior mean.

**Results:**

The heterogeneity of decision-makers in a population improved the overall benefit of treatment under some conditions.

**Discussion:**

In clinical settings, there exists heterogeneity of decision-making among patients. Our study investigated a targeting strategy by respecting the self-decision of all individuals and found that the heterogeneity of decision-making can improve the overall benefit under some conditions. In addition, this outperformance may suggest that heterogeneity of decision-making is of importance to human beings. Besides the ethical merit, our findings provide meaningful ideas for better strategies towards decision-making dilemmas in clinical settings for rare diseases or cases where only limited information is available. Furthermore, it is suggested to investigate the effects of heterogeneity of decision-making in other fashions, such as genetic heterogeneity and phenotypic heterogeneity.

## Introduction

The Randomized Controlled Trial (RCT) is considered to be the gold standard for the evaluation of treatment effects in medical settings. Since the design that combines randomization and blinding in the RCT minimizes selection bias and distributes confounders between the placebo group and the intervention group, the assessment of outcomes is more objective and accurate (Abel & Koch, 1999; Schulz & Grimes, 2002). While the classic RCT design is suitable for common diseases, it is less feasible in rare diseases because it is too time consuming to obtain a sufficient sample size (Edwards et al., 1997; Wilcken, 2001; Gerss & Kopcke, 2010). Moreover, the outbreak of acute lethal diseases requires patients to select one option among few with limited information. A typical example of this was the Ebola infection outbreak of 2013–2016, when empirical treatments were offered to patients without RCT. Parents may be reluctant to enrol their child in a placebo-controlled trial where he or she may receive a placebo rather than undergo the intervention of a treatment (Nature Editorial Board, 2010). In fact, there are precedents for approval of orphan drugs treating rare neurological diseases based only on pilot studies using smaller trial sizes and without the RCT principles (Mitsumoto et al., 2009).

Here, we assume a medical condition in practice. Two new treatments are developed for a rare disease with a high lethality rate; however, there is no or very limited evidence for both treatments’ effect in the current status. Under these conditions, patients themselves are disposed to try one of the two treatments rather than waiting for death. As we do not know the true effects (the true success rate) of either treatment, the very first patient will select either treatment with probability 0.5, that means she or he is assigned to the treatment with a lower success rate with probability 0.5. With the increase of sample size, strong evidence would eventually be obtained to clarify which treatment is superior to the other. However, in the process, many of the benefits to the patient are sacrificed, in particular individuals who are enrolled in the earlier stages of the trial. Although we investigated the effects of heterogeneity of decision makings, it is important to state that we do not mean the heterogeneity-oriented approach should replace the well-established approaches for clinical trials where random assignment is critical to assure the results of the studies. Our objective is to study the heterogeneity of decision makings and its effects in more general settings.

One of the classic Bayesian decision rules is to take the treatment with the largest posterior maximum expected success rate encoded in the posterior distribution. Then, patients are assigned to the treatment with the largest posterior maximum expected success rate (James, 1985; Donald & Bert, 1985). However, this causes a problem. While a single patient is assigned to one out of two treatments with a higher posterior mean, it prevents exploration of the other treatment, which may be the true superior. Thus, the problem is the dilemma between exploitation (enjoying the benefit of using the current knowledge) and exploration (taking the risk of obtaining new knowledge) (Berger-Tal et al., 2014). Many studies in statistical decision theory have revealed that the validity of the loss function-based approach can quantitatively demonstrate the optimal decision strategy and its limitation when information is limited (Savage, 1972; Bernardo & Smith, 1994). In addition to the statistical decision theory, there is another important aspect in clinical decision-making by patients. As we clinicians see patients, we find them heterogeneous regarding selection among options. Some aspects of the heterogeneity seem to be explained by inadequate understanding of statistical information on the options. Some aspects of the heterogeneity seem to be explained by the heterogeneity of personalized conditions or weighting parameters of loss functions. In addition, however, there seems to be heterogeneity in risk-taking. One such example is the attitude towards clinical trials. Some patients are very positive towards them, and some decline the idea, while others waver somewhere between. We considered that the risk-takers tend to see the optimistic aspects of the unknowns and the risk-hesitaters tend to see the pessimistic aspects.

Because we were interested in whether patient optimism/pessimism heterogeneity could mitigate the problem of the exploration-exploitation dilemma, we designed this study to evaluate the effects of heterogeneity of decision making on the overall success rate of treatments in a population. In the study, we generated a simple decision-making model with the heterogeneity. The heterogeneity of decision-making was parameterized for optimism/pessimism, called as the targeting decision strategy (T-strategy). With the Bayesian decision-theoretic approach (Bernardo & Smith, 1994), patients’ belief in the current state of knowledge regarding the success rate of each treatment is estimated from the Beta posterior distribution. Then, patients taking the T-strategy will select the treatment with the higher posterior probability that success rate is more than a targeting value. This value is calculated from a function of the larger posterior mean of success rate, but depending on the patient’s attitude. To evaluate the effects of T-strategy, we compared it with one classic decision rule which is defined as selecting the treatment with the larger posterior mean only, called as E-strategy. Using simulated datasets with two new treatments options, we compared the overall success rate of two treatments based on some conditions between the patients who are taking T-strategy and E-strategy separately, and quantitated the effects of the heterogeneity of decision-making.

## Materials & Methods

### Context

In this paper, we study the two-armed-bandits problem in clinical settings in which there are two new treatments for a disease without knowledge of success, denoted *A* and *B*. Each patient’s Bernoulli outcome, favourable (success) or unfavourable (failure), is recorded. The true rates of favourable outcomes for *A* and *B* are unknown, and they are denoted *a* and *b*, with 0 < *a*, *b* < 1. A series of patients select *A* or *B*, one by one, and the next (*n* + 1)^*th*^ patient is informed with the preceding outcomes that *n* patients have been treated in total and *n*_*A*_ and *n*_*B*_ have selected *A* and *B*, respectively, with *n*_*As*_ and *n*_*Bs*_ successful outcomes and *n*_*Af*_ and *N*_*Bf*_ failures, respectively. As shown in Table 1, *n* = *n*_*A*_ + *n*_*B*_, *n*_*A*_ = *n*_*As*_ + *n*_*Af*_, *n*_*B*_ = *n*_*Bs*_ + *n*_*Bf*_, *n*_*s*_ = *n*_*As*_ + *n*_*Bs*_, and *n*_*f*_ = *n*_*Af*_ + *n*_*Bf*_.

**Table 1:** Bernoulli outcomes for two treatments after *N* decision processes.

	**Favorable outcome**	**Unfavorable outcome**	
*A*	*N*_*As*_	*N*_*Af*_	*N*_*A*_
*B*	*N*_*Bs*_	*N*_*Bf*_	*N*_*B*_
	*N*_*s*_	*N*_*f*_	*N*

**Notes.**

*N*_*As*_ and *N*_*Af*_ are favorable (successes) and unfavorable (failures) outcomes of patients who selected treatment A. Correspondingly, *N*_*Bs*_ and *N*_*Bf*_ are favorable and unfavorable outcomes of patients who selected treatment B.

### Beta conjugate distribution to binomial outcomes

The outcome of each treatment is a Bernoulli outcome, and the parameter of unknown success rate follows a binomial distribution. In Bayes’ theorem, whereby the posterior is proportional to the prior multiplied by likelihood, there is one advantage in that the beta distribution is the conjugate distribution to Binomial outcomes (shown as Note S1). When no patient has been treated, we set a uniform Beta(1,1) as the initial prior, and then the two parameters of the prior are to be updated by the outcomes, successes *n*_∗*s*_ + 1 and failures *n*_∗*f*_ + 1.

(1) \begin{eqnarray*}{p}_{\mathrm{\ast }}(\theta {|}{n}_{\ast s},{n}_{\ast f})=\beta \left( {n}_{\ast s}+1,{n}_{\ast f}+1 \right) = \frac{1}{\mathrm{B}({n}_{\mathrm{\ast }s}+1,{n}_{\mathrm{\ast }f}+1)} {\theta }^{{n}_{\mathrm{\ast }s}}(1-\theta )^{{n}_{\mathrm{\ast }f}},\end{eqnarray*}

where ∗indicates *A* or *B*, *β* and B indicate the beta distribution and function, respectively, and 0 ≤ *θ* ≤ 1.

### Optimistic/pessimistic individuals in a population with heterogeneity of decision-making

We assumed that every individual selects one out of two treatments (*A* or *B*) with a higher value that is estimated from the beta posterior distribution with given current outcomes (successes and failures) of each treatment, *p*_∗_(*θ*|*n*_∗*s*_, *n*_∗*f*_) (Eq. 1). In this study, we modelled two types of individuals; one type of individual selects a treatment based on the posterior mean *v*_∗*E*_ (Eq. 2). They select the treatment with the larger posterior mean/larger maximum expected success rate (Eq. 5). We call this type of individuals’ selection as the E-strategy, where E stands for “Expected”. The other type of individual is somehow optimistic or pessimistic and selects the treatment with a higher value that is different from the expected value and depends on each individual’s optimistic/pessimistic preference. We set an attitude index, *w*, to parameterize the two preferences of individuals, where the *w* of pessimistic individuals ranges from −1 to 0 and the optimistic *w* ranges from 0 to 1. In fact, in terms of treatment assignment in clinical settings, we assumed that optimistic individuals care whether the treatments are adequately successful or not and that they set a target value (*t*) higher than the maximum posterior mean (*v*_∗*E*_) (Eq. 4), and calculate the probability that success rate is higher than the *t*. This probability is denoted by ${v}_{\ast T} \left( t \right) $ (Eq. 3). By contrast, we assumed that pessimistic individuals set *t*lower than the maximum posterior mean (*v*_∗*E*_), and calculate the probability that success rate is higher than *t*. The modelled *t* is calculated from a function of maximum posterior mean (*v*_max*E*_) but depending on this individual’s attitude index *w* (Eq. 4). Those individuals who are optimistic or pessimistic select the treatment with higher *v*_∗*T*_ (Eq. 5). We refer to this type of individual’s decision as a T-strategy, where T stands for “Target”.

The posterior mean of the success rate is

(2) \begin{eqnarray*}{v}_{\mathrm{\ast }E}=\int \nolimits \nolimits _{0}^{1}\theta \times {p}_{\mathrm{ \ast }}(\theta {|}{n}_{\ast s},{n}_{\ast f})d\theta = \frac{ {n}_{\mathrm{\ast }s}+1 }{{n}_{\mathrm{\ast }s}+{n}_{\mathrm{\ast }f}+2} ,\end{eqnarray*}

where ∗ indicates *A* or *B*

The probability of a success rate more than a target value is

(3) \begin{eqnarray*}{v}_{\ast T}(t)=\int \nolimits \nolimits _{t}^{1}{\mathrm{p}}_{\ast } \left( \theta {|}{n}_{\ast s},{n}_{\ast f} \right) d\theta ,\end{eqnarray*}

where ∗ indicates *A* or *B*, and the target value *t* is calculated by *v*_*maxE*_ = *max*(*v*_*AE*_, *v*_*BE*_) and depends on the attitude index *w*.

(4) \begin{eqnarray*}t= \left\{ \begin{array}{@{}l@{}} \displaystyle \mathrm{w}+ \left( 1-\mathrm{w} \right) {v}_{maxE}, 0\leq \mathrm{w}\leq 1 \\ \displaystyle \left( 1+\mathrm{w} \right) {v}_{maxE}, -1\leq \mathrm{w} \lt 0 \end{array} \right. \end{eqnarray*}

where positive and negative *w* stands for optimism and pessimism, respectively. Correspondingly, the *v*_*maxE*_ ≤ t ≤ w specifies optimism, and 0 ≤ t ≤ *v*_*maxE*_ specifies the pessimism.

Subsequently, the probability of selecting *A*, *Prob*(*A*), is given as

(5) \begin{eqnarray*}Prob(A)= \left\{ \begin{array}{@{}l@{}} \displaystyle 1: {v}_{A\mathrm{\ast }}\gt {v}_{B\mathrm{\ast }} \\ \displaystyle 0.5: {v}_{A\mathrm{\ast }}={v}_{B\mathrm{\ast }} \\ \displaystyle 0: {v}_{A\mathrm{\ast }}\lt {v}_{B\mathrm{\ast }} \end{array} \right. ,\end{eqnarray*}

where ∗ indicates E or *T*. Actually the selection of every individual is deterministic based on the values that are calculated from the 2 by 2 table values in principle. The selection is stochastic only when the values are equal. When we assumed a population was homogeneous, their selection was deterministic except for the stochastic selection due to the identical values for two arms. When we assumed a population was heterogeneous, the individuals’ optimistic/pessimistic attitude vary among them and the sequence of individuals were stochastically generated in the experiment.

We assumed that the population is a mixture of individuals with various levels of optimism/pessimism. To specify the heterogeneous population in this model, we assumed that need w is symmetric around zero and is in a monomodal distribution ranging from -1 to 1. With this assumption, the majority of people are almost neutral and relatively few people are strongly optimistic or pessimistic. As a simple model for this distribution, we assumed

(6) \begin{eqnarray*}w\sim 2 \left( \beta \left( u,u \right) -0.5 \right) ,\end{eqnarray*}

where *u* parameterizes the shape of distribution of *w*.

**Figure 1:**
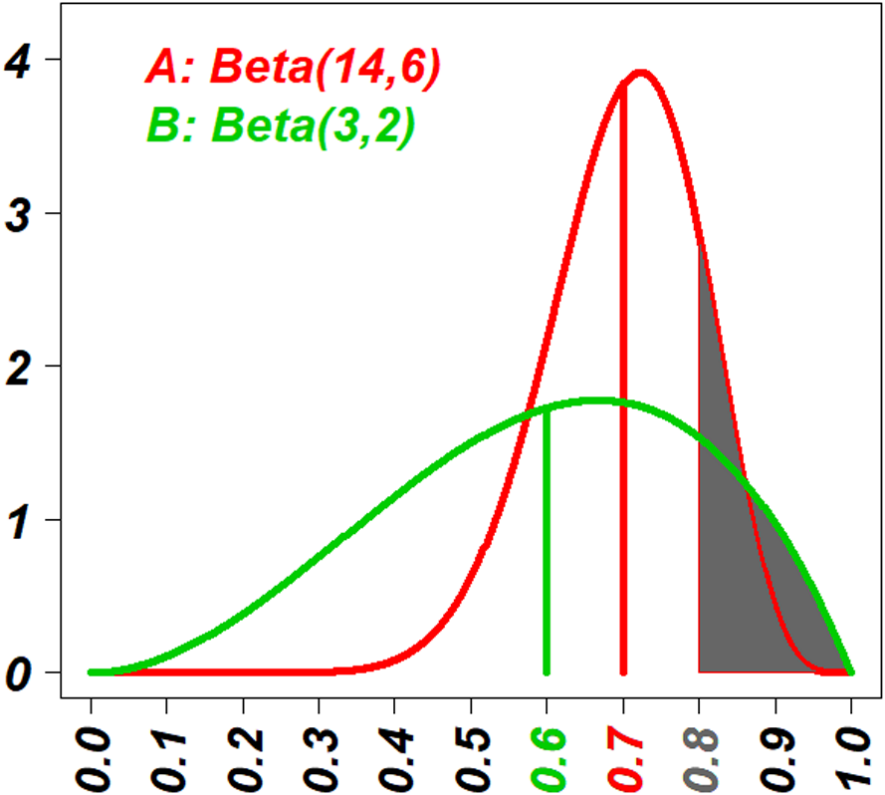
The visual explanation of decision values of E-strategy and T-strategy (*v*_∗*E*_ and *v*_∗*T*_). The red curve shows the probability density function (PDF) of the beta posterior distribution of the success rate of treatment *A* with 13 successes and five failures; and the green curve shows one of the treatment *B* with two successes and one failure. The red and green vertical lines indicate the posterior means of two distributions, *v*_*AE*_ and *v*_*BE*_, respectively. Actually *v*_*AE*_ = 0.7 and *v*_*BE*_ = 0.6. The vertical line that demarcates the gray areas under the curves indicates the target value, *t* = 0.8. The gray areas under the curves indicate the probability that their success rates are higher than the target value *t* = 0.8 for the two strategies, *v*_*AT*_ and *v*_*BT*_. Actually *v*_*AT*_ = 0.16 and *v*_*BT*_ = 0.18. Because *v*_*AE*_ >  *v*_*BE*_, people with E-strategy will select the treatment *A* and because *v*_*AT*_ < *v*_*BT*_, people with T-strategy will select the treatment *B*.

An example for the selection was given in the Fig. 1, where the Beta posterior distributions of two treatments were drawn with the information: the eighteen (18 = (14 − 1) + (6 − 1)) patients have been treated with the treatment *A*, resulting in outcomes of 13 successes and five failures, and three (3 = (3 − 2) + (2 − 1)) patients have been treated with *B*, resulting in outcomes of two successes and one failure. Two sets of decision values based on E-strategy (*v*_∗*E*_) and T-strategy (*v*_∗*T*_) were calculated separately, and in the Fig. 1 *v*_∗*E*_ are indicated as vertical lines (*v*_*AE*_ = 0.7 and *v*_*BE*_ = 0.6), and *v*_∗*T*_ are indicated as the area under the curve truncated by a vertical line (${v}_{AT} \left( t \right) =0.16$ and ${v}_{BT} \left( t \right) =0.18$). Since 0.7 > 0.6, individuals with E-strategy should select *A*. Since 0.16 < 0.18, individuals with T-strategy (*t* = 0.8 corresponds to an optimistic individual with *w* approximately 0.3) should select *B*.

### Experimental conditions

The true but unknown success rate of the two arms, *a* and *b*, and the number of total patients, *N*, are parameterized. The total 5,050 pairs of combination of *a* and *b* was generated with 0 < *b* ≤ *a* < 1. For the T-strategy population, *w* as an attitude index is parameterized within the range of −1 and 1. In addition, we evaluated relatively small *N* values, from 1 to 100, because our objective is to study the effects of optimism/pessimism of decision-makers for treatment assignment with a rarer disease in a clinical setting where the patient population size is small. Under the same condition of *a*, *b*, and *N*, we first compared the overall success rate between the homogeneous population who take the E-strategy and the homogeneous population who take the T-strategy, where all T-strategy individuals have the same *w* values.

After the comparison between the homogeneous E-strategy population and the homogeneous T-strategy population, we investigated the effects of heterogeneity. We generated a population with the T-strategy whose *w* values were not same but distributed as shown in Eq. (6), where *u* was 5, 30, 70 and 500.

### Measure of the overall benefit

#### Calculation of the exact probability of every Bernoulli outcome of the two arms

In this study, we enumerated all possible combinations of successes and failures for each treatment as a 2 × 2 table (shown as Note S2); then, the exact probability of every table consisting of *N*_*As*_, *N*_*Af*_, *N*_*Bs*_, and *N*_*Bf*_ was calculated as the below equation.

\begin{eqnarray*}& & Pr \left( {N}_{As},{N}_{Af},{N}_{Bs},{N}_{Bf} \right) \end{eqnarray*}

\begin{eqnarray*}& &  =Prob \left( A{|} \left( {N}_{As}-1,{N}_{Af},{N}_{Bs},{N}_{Bf} \right) \right) \times a\times Pr \left( {N}_{As}-1,{N}_{Af},{N}_{Bs},{N}_{Bf} \right) \end{eqnarray*}

\begin{eqnarray*}& &  +~Prob \left( A{|} \left( {N}_{As},{N}_{Af}-1,{N}_{Bs},{N}_{Bf} \right) \right) \times (1-a)\times Pr \left( {N}_{As},{N}_{Af}-1,{N}_{Bs},{N}_{Bf} \right) \end{eqnarray*}

\begin{eqnarray*}& &  +~ \left( 1-Prob \left( A{|} \left( {N}_{As},{N}_{Af},{N}_{Bs}-1,{N}_{Bf} \right) \right) \right) \times b\times Pr \left( {N}_{As},{N}_{Af},{N}_{Bs}-1,{N}_{Bf} \right) \end{eqnarray*}

(7) \begin{eqnarray*}& & +~ \left( 1-Prob \left( A{|} \left( {N}_{As},{N}_{Af},{N}_{Bs},{N}_{Bf}-1 \right) \right) \right) \times (1-b)\times Pr \left( {N}_{As},{N}_{Af},{N}_{Bs},{N}_{Bf}-1 \right) \end{eqnarray*}

when patient number *N* = 0, then $Pr \left( 0,0,0,0 \right) =1.$

For heterogeneous decision-makers, we assigned w to a series of patients from the distribution in the Eq. (6) with the indicated u value. Because the stochastic processes vary with the sequence of *w* values, we iterated 3,000 random Monte Carlo patient sequences (Kroese et al., 2014) up to *N* = 50, and we calculated the average of $Pr \left( {N}_{As},{N}_{Af},{N}_{Bs},{N}_{Bf} \right) $.

#### Measure of the overall success rate

We emphasized the evaluation of the overall benefit of treatments in a population rather than an individual’s best benefit; thus, we measured the average fraction of favourable outcomes (successes) for the series of *N* patients as a whole when *a* and *b* were given regardless of the selected arm, named the Overall Success Rate (OSR).

(8) \begin{eqnarray*}OSR \left( N \right) =\sum _{ \left\{ \left( {N}_{As},{N}_{Af},{N}_{Bs},{N}_{Bf} \right) {|}{N}_{As}+{N}_{Af}+{N}_{Bs}+{N}_{Bf}=N \right\} } \frac{{N}_{As}+{N}_{Bs}}{N} Pr \left( {N}_{As},{N}_{Af},{N}_{Bs},{N}_{Bf} \right) .\end{eqnarray*}

All calculation was performed with the R, and the code is available at the following URL: https://github.com/statgenetJimu/SelfDecABP/blob/master/SelfDecABP(1).%20r-package.

## Results

First, we showed a typical case of homogeneous E-strategy (*E*.*st*) and homogeneous T-strategy (*T*.*st*). Second, we demonstrated the detailed effects of conditions of *a*, *b* and *N* on the difference between homogeneous *E*.*st* and homogeneous *T*.*st*. Third, we showed the benefit of the heterogeneous *T*.*st*.

### Typical case of homogeneous E-strategy (*E*.*st*) and homogeneous T-strategy (*T*.***st***)

**Figure 2:**
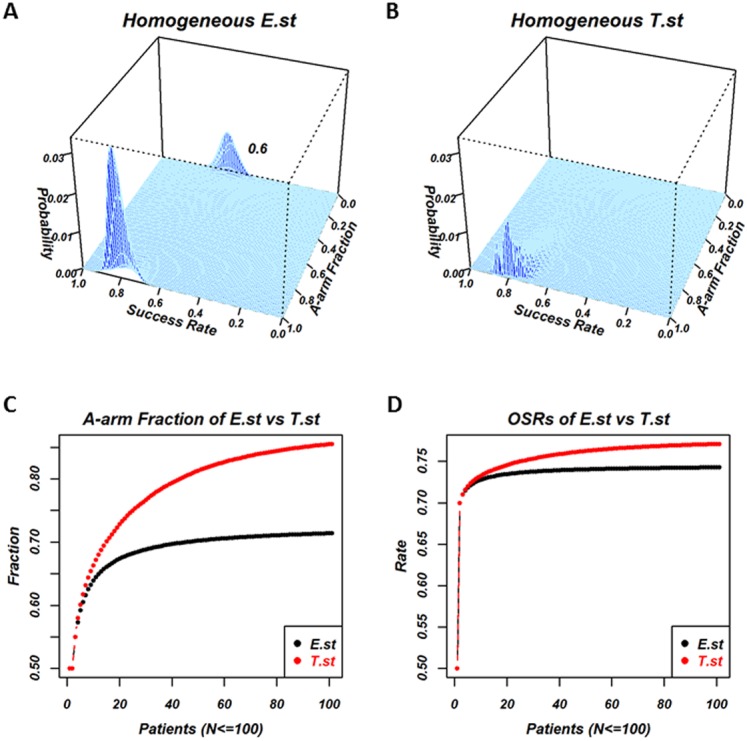
Comparison of a homogeneous population with E-strategy (*E.st*) and a homogeneous population with T-strategy (*T.st*) when the true success rates of A and B were 0.8 and 0.6 and the optimistic attitude index of *T.st* was 0.5. (A) and (B) show the results when 100 patients’ outcomes have been recorded for *E.st* and *T.st*, respectively. Each panel is the two-dimensional histogram where the cyan area indicates the support plane and one axis “Success rate” indicates the overall success rate for 100 patients and the other axis “A-arm Fraction” indicates the fraction of patients who selected treatment *A*. The vertical axis indicates the exact probability of occurrence in the stochastic process. (A) for *E.st* shows two peaks; one peak’s overall success rate was around 0.8 and its A-arm fraction was close to 1 and the other peak’s overall success rate was around 0.6 and its A-arm fraction was close to 0. The first peak was higher than the other peak. These findings indicated that the majority of individuals with *E.st* selected treatment *A* in many occasions but that in some occasions, they selected treatment *B* rather than treatment *A*. (B) for *T.st* shows one peak and its overall success rate was around 0.8 and its A-arm fraction was close to 1. The mountain in (B) was lower than the mountain located nearby in (A). These findings indicated that the majority of individuals with *T.st* selected treatment *A* in almost all the occasions, although the A-arm fraction tended to be lower than *E.st.* (C) and (D) show how the two measures change based on the homogeneous *E.st* versus *T.st* while the patient number changed, *N* = 1, 2, …, 100, and horizontal axis shows the patient’s number, and vertical axis shows the measure “A-arm fraction” in the (C) and the measure “overall success rate (OSR)” in the (D), where homogeneous *E.st* is labeled in black and *T.st* is in red.

Individuals in the homogeneous *E*.*st* population select treatments based on the posterior mean, *v*_∗*E*,_. Individuals in the homogeneous *T*.*st* population select treatments based on *v*_∗*T*_, which represents the optimism/pessimism attitude and is shared by all the individuals in the population. Figure 2 is the results of the experiment, where the success rate of the two treatments were *a* = 0.8 and *b* = 0.6,  andthe optimistic attitude *w* of the *T*.*st* population was 0.5. The total number of patients was *N* = 100. Figures 2A and 2B are the 2-dimensional histograms, where one axis is the fraction of individuals who selected *A* and the other axis is the overall success rate when all the processes reached *N* = 100 for *E*.*st* and *T*.*st*, respectively. The processes of selection by a series of individuals are stochastic; the fraction of selecting *A* and the overall success rate take distribution. The exact distribution of the fraction and the rate were calculated and displayed. In the case of *E*.*st*, as shown in Fig. 2A, the distribution was bimodal, with the higher peak corresponding to the occasions in which a majority of *N* patients had selected the better treatment arm, *A*, and the lower peak indicating that the minority had selected the inferior treatment arm, *B*, with a subsequently lower OSR. In the case of *T*.*st*, as shown in Fig. 2B, the distribution was monomodal with the peak towards the *A*-arm selection. The bimodality was the result of the exploitation-exploration dilemma. In some cases, the patients who selected the inferior one turn out to be successful with the expected success rate higher than the true success rate because this is a stochastic process. In this case, the following patients tend to select the inferior treatment arm with the belief that this treatment arm has a high success rate, and they lose the chance to select the other treatment arm that was truly better. Panels A and B indicate that the decision strategy of the population affected the exploitation-exploration pattern.

Figures 2C and 2D show the average fraction of individuals who selected the *A*-arm and the OSRs among a number of patients from 1to 100 of the two strategies, respectively. Panel C shows that the A-arm fraction of *E*.*st* and *T*.*st* at *N* = 100 was 0.714 and 0.855, respectively, and the fraction was higher for *T*.*st* throughout for the number of patients. Panel D shows that OSR of *E*.*st* and *T*.*st* at *N* = 100 was 0.743 and 0.771, respectively, and the rate was higher for *T*.*st* throughout. This finding was also related to the lack of exploration that occurred in *E*.*st* in this particular scenario.

### Comparison of overall benefit of homogeneous decision-makers between E-strategy (*E*.*st*) and T-strategy (*T*.*st*) with the same attitude index *w* value

The typical case above showed that the homogeneous decision-makers of *T*.*st* with *w* = 0.5 outperformed *E*.*st* when *a* = 0.8 and *b* = 0.6 for *N* = 1, 2, …, 100. However, such superiority is not always true for all conditions of *a*, *b*, *N*,  and *w*. In fact, under some conditions, homogeneous *T*.*st* decision-makers outperformed the homogeneous *E*.*st*, but under other conditions, the *E*.*st* decision-makers outperformed the *T*.*st*.

We evaluated the difference of Overall Success Rates (OSRs) between homogeneous *T*.*st* and homogeneous *E*.*st* decision-makers with the same attitudes of *w* values for various conditions; *N* = 1, 2, …, 100 and $ \left( a,b \right) = \left\{ \left( a,b \right) {|}a,b\in \left\{ 0.01,0.02,\ldots ,0.99 \right\} ,a\geqq b \right\} ,w=${−0.0005, −0.001, …, 0.999, 0.9995}. The number of (*a*, *b*) pairs was 5,050.

**Figure 3:**
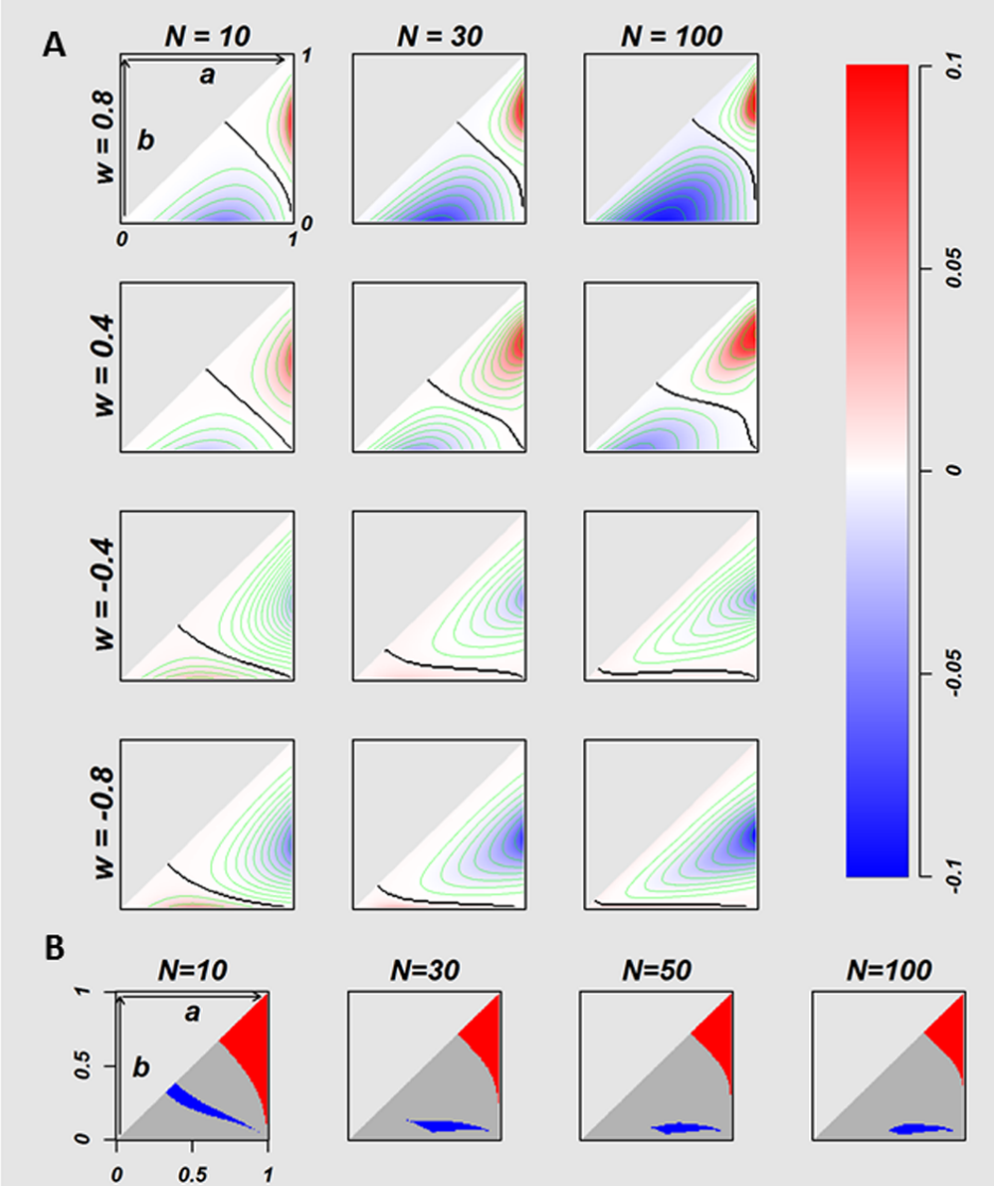
The relation between true success rates of two treatments and overall success rates of two strategies. The difference of the OSRs (*T.st –E.st*) between homogeneous *E.st* decision-makers and homogeneous *T.st* decision-makers with the fixed attitude *w* values, on the conditions where 5,050 of true success rates pairs ((*a*, *b*) = {(*a*, *b*)|*a*, *b* ∈ {0.01, 0.02, …, 0.99}, 1 > *a* ≥ *b* > 0}) were calculated for multiple *w* values of *T.st* population (*w* = 0.8, 0.4,  − 0.4, and −0.8). The twelve plots in the (A) indicate *N* = 10, 30 and 100 for every *w* values. Each plot has a triangle area that corresponds to the (A, B) pairs where a ≥*b*. The negative and positive values of difference of OSRs are coded in blue and red, respectively. The black curves in the triangle areas indicate the (A, B) pairs without difference in OSRs between two strategies. In the (B) the red indicates the (A, B) pairs where the optimistic homogeneous *T.st* decision-makers performed better than *E.st* with regardless of the optimistic *w* values where the number of the fixed *w* is 2,000, with *w* ∈ 0, …, 0.999, 0.9995, the blue indicates the area of (A, B) pairs where *E.st* performed better, and the gray indicates the (A, B) pairs where the *T.st* or *E.st* performed better with depending on the *w* value.

Figure 3A visualizes the difference of OSR of 5,050 (*a*, *b*) pairs at *N* = 10, 30,  and 100 for *w* =  − 0.8,  − 0.4, 0.4, and 0.8. The (*a*, *b*) pairs form a triangular space. The horizontal axis is *a*, and the vertical axis is *b*. Red indicates (*a*, *b*) pairs where *T*.*st* outperforms *E*.*st*, and blue indicates (*a*, *b*) pairs where *E*.*st* outperforms *T*.*st*. Colour intensity stands for the value of the difference of OSRs as indicated in the colour bar on the right. The black curves stand for the (*a*, *b*) pairs without difference between *E*.*st* and *T*.*st*.

The colour patterns of 12 conditions of the Fig. 3A show that the superiority of two strategies is the function of (*a*, *b*) conditional to *N* and *w*. The homogeneous optimistic decision-makers (*T*.*st* with *w* > 0) outperformed *E*.*st* when both *a* and *b* were relatively large, but their performance was worse than *E*.*st* when both *a* and *b* were relatively small, as shown in the first and second rows of *w* = 0.4 and 0.8 of Fig. 3A. When N increases, the colour intensity tends to become stronger. In contrast, the performance of the homogeneously pessimistic attitude decision-makers (*T*.*st* with *w* < 0) was worse than *E*.*st*for the majority of (*a*, *b*) pairs, and *T*.*st* outperformed only when both a and b were small. When *N* increases, the colour intensity tends to become stronger.

Next, we investigated the relation between (*a*, *b*) pairs and the superiority of *E*.*st* and *T*.*st*, regardless of the intensity of optimism or regardless of *w* values as far as *w* > 0. We calculated OSRs for *w* = {0, …, 0.999, 0.9995}. Fig. 3B coloured the (*a*, *b*) pairs with red, grey and blue, where red indicates that the average OSR of *T*.*st* is higher than the OSR of *E*.*st* for all optimistic *w* values, and blue indicates that the average OSR of *T*.*st* is lower than the OSR of *E*.*st* for all optimistic *w* values, and grey indicates otherwise. In general, *T*.*st* tends to outperform when both treatments have a relatively high success rate and *E*.*st* outperforms when both treatments have a relatively low success rate. In this comparison, we evaluated homogeneous *T*.*st* populations from which all individuals in a population were the same *w* values, and we set different such same *w* (ranging from −1 to 1) values for each of homogenous populations. In the next experiment, we modelled populations that are heterogeneous for decision attitudes that consisted of individuals whose optimism/pessimism attitude index *w* varied and compared their performance with *E*.*st*.

**Figure 4:**
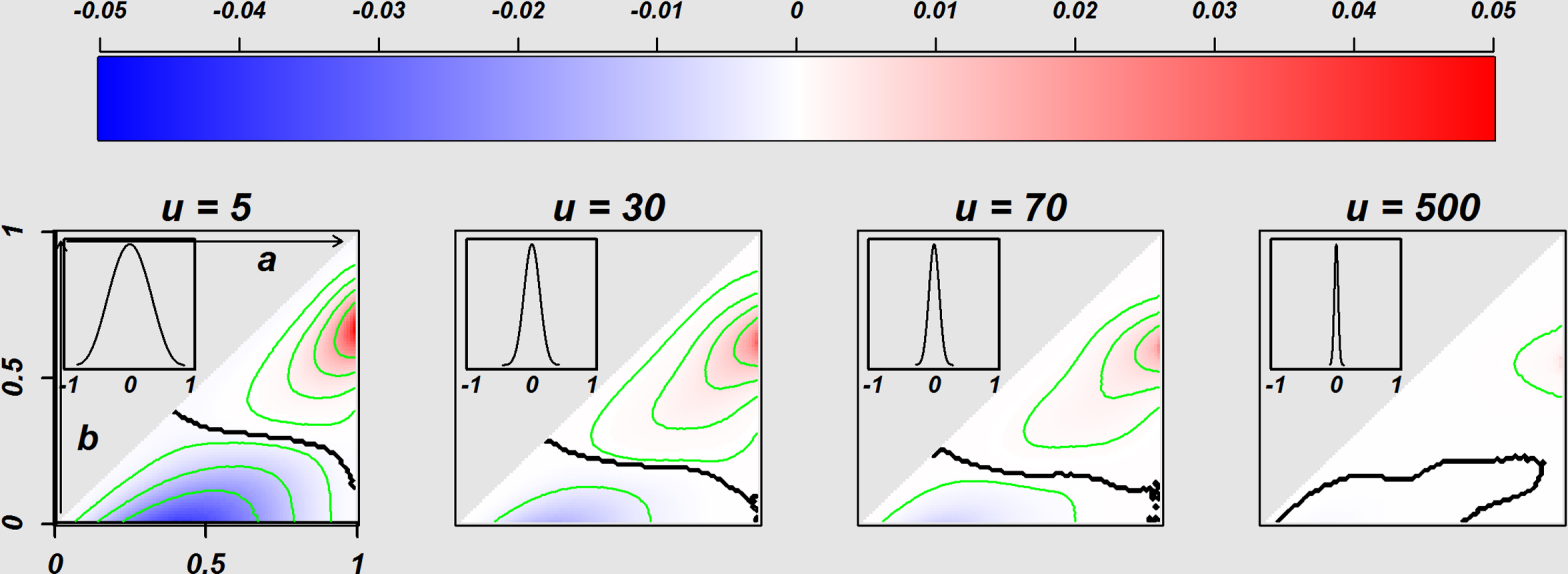
Comparison of the Homogeneous *E.st.* population and Heterogeneous *T.st* population. Figure 4 indicates the difference of the OSRs between the decision-makers in a population with heterogeneity of decision attitudes (*w* values of individuals are various) and the homogeneous *E.st* decision-makers. The four panels separately indicate the difference of OSRs based on the four distributions of attitudes with given various beta parameter *u* values of 5, 30, 70, and 500 separately, where the distribution of *w* is located on the top-left of each image (*w* values were generated from the distribution shown as the (6) in the methods section). The triangle area in each image corresponds to 5,050 success rate pairs (*a*, *b*) = {(*a*, *b*)|*a*, *b* ∈ {0.01, 0.02, …, 0.99}, *a*≧*b*}, and the difference of OSRs in the each triangle was coded with blue and red with representing negatives and positives, respectively. The sample size is *N* = 50. Such OSRs for the heterogeneity population were the average of 3,000 Monte Carlo iterations.

### The effect of heterogeneity of decision-makers in a population

In reality, populations seem to consist of individuals with various attitudes. We modelled heterogeneity of optimism/pessimism attitude index *w* with Eq. (6), where *w* distributes symmetric around 0. Figure 4 is the colour plot to display the superiority of *E*.*st* andheterogeneous *T*.*st*. Four panels show the result of four different distributions of optimism/pessimism index *w*, specified with *u* = 5, 30, 70 and 500. The distribution of *w* is displayed in a window of each panel.

Figure 4 show some tendencies. The heterogeneous *T*.*st* tended to outperform *E*.*st* (red) when both *a* and *b* were relatively high. With smaller *u* or larger variance of *w*, the difference of OSR was bigger. Actually, the right most panel indicates essentially no difference between *E*.*st* and heterogeneous *T*.*st* when the variance of w is very small. When *u* is small (u=5), the triangular area was divided into two coloured subareas almost evenly, and when u is larger (*u* = 30 and 70), the area where the heterogeneous *T*.*st* was superior was bigger than the area where *E*.*st*was better.

## Discussion

We assumed that decision-makers might vary in a population due to the heterogeneity of individuals’ decision attitudes in medical decision-making. In reality, it is ethical to respect individuals’ self-decision particularly when there is no sufficient information to make decisions for certain. In clinical settings, these conditions correspond to rare diseases or patients with a common disease that is complicated by various conditions, where some information is available but is not conclusive. If all individuals of a population are E-strategy decision-makers, the outcome for the whole population may not be optimal, which is the result of the trade-off between exploitation and exploration where an individual selects one arm to optimize the outcome for herself/himself but the population loses the chance to take another arm that might be better; this is the exploration-exploitation dilemma and the multi-armed bandit problem (Berger-Tal et al., 2014; Robbins, 1952; Auer, Cesa-Bianchi & Fischer, 2002; Press, 2009). This phenomenon was shown in Fig. 2. Although statistical studies on the multi-armed bandit problem developed decisions to optimize the outcome of the whole population strategies, such as Gittin’s index (Gittins, 1979; Gittins & Jones, 1979; Karoui & Karatzas, 1993), they did not respect individuals’ self-decisions. Thus, we studied the effects of heterogeneity of decision-making on the overall benefit of the whole population in a simple clinical setting. Although we stated in the introduction section, it should be stated again that we have investigated the possible benefit in heterogeneity of decision makings in population but that we do not mean that patients should select a treatment from multiple options and the importance of clinical trials and the evidence-based medicine is the gold standard in clinics.

The question here is whether we, as humans, are truly heterogeneous in our selections. It is true the individuals’ background heterogeneity, such as comorbidity, cost, life-style and age and so on, in case of clinical conditions, can cause their decisions heterogeneous and these hidden factors might explain all the components of heterogeneity of decision making among people. However it is also true that we, clinicians, face the heterogeneous attitudes among patients that do not seem to be well explained by the factors. The particular example of this heterogeneity is the attitude heterogeneity towards clinical trials. We recruit patients for a clinical study who are homogeneous enough to meet inclusion/exclusion criteria, and some patients participate in it and others decline the idea. We believe the enrollment or no-enrollment should not be heavily biased with hidden factors and we are not sure why some participate and some not. We investigated this unclear heterogeneity in our study. Another example of the heterogeneity of decision makings among people can be seen in attitude towards gambles. It is obvious that nobody should anticipate gain as a whole, but still some people keep gambling and some won’t. Therefore the assumption of heterogeneity of decision making seemed reasonable to be investigated. In this study we modelled the heterogeneity in decision makings very simply. When two treatments have never been applied and someone is the first patient to be treated with either of one of the two, he or she will select either treatment with a probability of 0.5. When one treatment has been used18 times with 13 favourable and five unfavourable outcomes and the other treatment has been subjected to three attempts with two favourable and one unfavourable outcome, which treatment would be selected? If you ask this question to many people without further information, their answers would vary. If you add the information with the knowledge of the posterior mean and the two treatments are (13 + 1)∕(13 + 1 + 5 + 1) = 14∕20 = 0.7 and (2 + 1)∕(2 + 1 + 1 + 1) = 3∕5 = 0.6, then all or the vast majority would likely select the first treatment (shown in the red and green vertical lines, respectively in Fig. 1). In other words, some people who may initially prefer the secondtreatment with the information of (two out of 3) have to change their preference to select the first treatment with the information (13 out of 18) if depending only on the posterior mean. Is this change in preference due to the lack of “statistical literacy”? The answer could be yes or no. Given the information of 13 out of 18 of the first treatment, the distribution of the favourable outcome probability is a beta distribution with shape parameters 14 and 6 whose posterior mean is 0.7. Using the same distribution, the probability that the first treatment could have a success rate of more than 0.8 is 0.16 (shown in the grey areas under the red and green curve separately in the Fig. 1). Based on the outcome information of two treatments, we have two sets of values. One set is the expected success probability of two treatments, (the first treatment 0.7, the second treatment 0.6). The other set is the probability that success probability should be more than 0.8 (the first treatment 0.16, the second treatment 0.18). If we select the treatment with higher expected probability, we should use the value set, (the first treatment 0.7, the second treatment 0.6), and because 0.7 > 0.6, we should take the first treatment. If we select the treatment with higher probability that the first treatment could have a success rate of more than 0.8, we should use the value set, (the first treatment 0.16, the second treatment 0.18), and because 0.18 > 0.16, we should take the second treatment. Based on this hypothetical evaluation, it can be said that the individuals who selected the treatment with 2 vs.1 might bet on it because of its potential to be a truly good treatment. We call this attitude “optimistic”. In fact, when we design a clinical trial to test a newer treatment against a standard treatment, we should be optimistic enough to believe that there is some chance that the newer treatment might be better than the standard treatment. Based on this idea, we modelled a decision attitude, the T-strategy, which compared the likelihood that the success rates are higher than the targeted value that reflects optimism/pessimism and their intensity.

Through the comparison of the overall benefit in a population between the homogenous decision-makers of E-strategies and homogeneous decision-makers of T-strategies with fixed optimism/pessimism parameters, our study revealed the following: First, the optimistic homogeneous decision-makers of the T-strategy outperformed the E-strategy when the true success rates of both arms were relatively high, and the pessimistic homogeneous decision-makers of T-strategy performed best when the true success rates of both were relatively low. Second, the effects of optimism and pessimism were asymmetric. The area of the optimistic homogeneous decision-makers of *T*.*st* with better performance than that of *E*.*st* tended to be wider when compared with the pessimistic homogeneous decision-maker of *T*.*st* with better performance than that of *E*.*st*. Additionally, homogeneous optimism worked better regardless of the intensity of the optimism for some conditions, but homogeneous pessimism did not have any such conditions (Fig. 3). Furthermore, through the comparison of the overall benefit in a population between the homogenous decision-makers of E-strategies and the heterogeneous decision-makers with a symmetric mixture of optimists and pessimists of various intensities, our study revealed that the heterogeneous decision-makers in a population outperformed the homogeneous E-strategy when the true success rates of the two arms were relatively high. When the variation in optimism/pessimism was small, the degree of benefit was small, but the conditions *a* and *b* in which heterogeneity outperformed the homogeneity of the E-strategy decision-maker were wide and vice versa (Fig. 4). These findings suggested that when the success rates of two treatment arms for patients with rare diseases are believed to be relatively high, the decision-makers with an optimistic decision attitude in a population would be the best. In addition, due to the wider conditions of beneficial effects of the homogeneous optimistic than pessimistic attitudes, optimism should be encouraged over pessimism if the decision-makers are homogeneous. When the decision-makers in a population are heterogeneous, a large variation performs better under narrower conditions with stronger intension. Those findings are practical not only in medical decision-making but also in other fields of decision-making. For example, in a complex system in network science, recent studies in the field have reported that the heterogeneity of factors increased the structural vulnerability of the system (Sun et al., 2016). In the context of our study, the sequence of individuals can be considered to be a directed line graph in which information flows and the heterogeneity of the factors of the network is attributed to the heterogeneity of each individual.

In our study, we evaluated only a small population size by enumerating all combinations of outcomes of each treatment at each status by calculating the exact probabilities, each of which is formed as a 2 × 2 table pattern consisting of four integer numbers. For the larger population sizes, we evaluated the cases with *N* = 500 using the Monte–Carlo simulation methods (Kroese et al., 2014) rather than the exact probability calculation, which showed a qualitatively similar phenomena to the ones we observed for the smaller size. Because our investigation was limited to a very specific scenario and an artificial attitude model, further studies should be performed; the following seemed to be hypothesized. The benefit of a single individual will be maximized by selecting the option with the higher expected success rate. However, when all the individuals of a population take the same decision strategy, the overall benefit of the whole population may not be optimized in some cases. This is the phenomenon of the exploitation-exploration dilemma. When the individuals of the population are heterogeneous regarding decision-making, the dilemma seemed to be mitigated at least partially. In addition, this idea is compatible with the clinical scenario where patients’ self-decisions should be respected. Although we do not know whether human beings are heterogeneous in decision-making, it is possible because human beings are heterogeneous in many ways, such as genetically and phenotypically, and because the heterogeneity in various aspects is believed to be important for the sustainability of the species. One more interesting finding was that the optimistic attitude and the heterogeneity of optimism/pessimism performed better when both options had a higher success rate. Because all species including human beings participate in the survival game of evolutional history, they keep trying to find ways with higher success rates. Thus, it may be the case that the majority of selection tasks are selections among options with relatively higher success rates. If all of these assumptions are true, heterogeneity with some inclination towards the optimistic side could be one of the best strategies for populations. Again, these hypotheses were based on our limited investigations, and further studies are necessary.

Although the overall benefit of treating is improved if the heterogeneity of decision-making in a population is considered by respecting every individual’s decision attitude, in fact, other realistic factors might be combined in our proposed heterogeneity of decision-making, e.g., cost, life-style and age. Considering that there are far more complicated cases in real clinical works, it would require effective cooperation between statisticians and clinicians for further investigation when more factors are introduced into the heterogeneity model.

## Conclusions

We modelled the heterogeneity of decision-making in populations in terms of optimism and pessimism and compared them with the decision rule based on the expected success rate. We identified that the optimistic or pessimistic strategy outperforms the expected value-based strategy when success rates of options are in particular conditions. In addition, when a population consists of individuals with heterogeneous optimistic/pessimistic attitudes, it was able to outperform when it pursues options with a high success rate. This outperformance is achieved by respecting the self-decision of all individuals, which is ethically important. Our findings may provide meaningful ways to find better strategies for the decision-making dilemma in clinical settings for rare diseases or cases where only limited information is available. It is further suggested to investigate the effects of heterogeneity of decision-making in other aspects, such as genetic heterogeneity and phenotypic heterogeneity.

##  Supplemental Information

10.7717/peerj.5677/supp-1 Note S1 The proof of Beta conjugate to Bernoulli/Binomial distribution

10.7717/peerj.5677/supp-2 Note S2 The diagram of enumerating all possible 2x2 tables and exact probability calculation

## Additional Information and Declarations

### Data Availability

The following information was supplied regarding data availability: The exact probability calculation and the Monte Carlo simulation were performed with our R code, which is available at GitHub:
https://github.com/statgenetJimu/SelfDecABP/blob/master/SelfDecABP(1).%20r-package.

## References

[ref-1] Abel U, Koch A (1999). The role of randomization in clinical studies: myths and beliefs. Journal of Clinical Epidemiology.

[ref-2] Auer P, Cesa-Bianchi N, Fischer P (2002). Finite-time analysis of the multiarmed bandit problem. Mach Learn.

[ref-3] Berger-Tal O, Nathan J, Meron E, Saltz D (2014). The exploration-exploitation dilemma: a multidisciplinary framework. PLOS ONE.

[ref-4] Bernardo JM, Smith AFM (1994). Bayesian theory.

[ref-5] Donald AB, Bert F (1985). Bandit problems: sequential allocation of experiments.

[ref-6] Edwards SJ, Lilford RJ, Braunholtz D, Jackson J (1997). Why underpowered trials are not necessarily unethical. Lancet.

[ref-7] Gerss JW, Kopcke W (2010). Clinical trials and rare diseases. Advances in Experimental Medicine and Biology.

[ref-8] Gittins JC (1979). Bandit processes and dynamic allocation indices. Journal of the Royal Statistical Society: Series B.

[ref-9] Gittins JC, Jones DM (1979). A dynamic allocation index for the discounted multiarmed bandit problem. Biometrika.

[ref-10] James OB (1985). Statistical decision theory and Bayesian analysis.

[ref-11] Karoui NE, Karatzas I (1993). General Gittins index processes in discrete time. Proceedings of the National Academy of Sciences of the United States of America.

[ref-12] Kroese DP, Brereton T, Taimre T, Botev ZI (2014). Why the Monte Carlo method is so important today. WIREs Computational Statistics.

[ref-13] Mitsumoto J, Dorsey ER, Beck CA, Kieburtz K, Griggs RC (2009). Pivotal studies of orphan drugs approved for neurological diseases. Annals of Neurology.

[ref-14] Nature Editorial Board (2010). The needs of the few. Nature.

[ref-15] Press WH (2009). Bandit solutions provide unified ethical models for randomized clinical trials and comparative effectiveness research. Proceedings of the National Academy of Sciences of the United States of America.

[ref-16] Robbins H (1952). Some aspects of the sequential design of experiments. Bulletin of American Mathematical Society.

[ref-17] Savage LJ (1972). The foundation of statistics.

[ref-18] Schulz KF, Grimes DA (2002). Blinding in randomized trials: hiding who got what. Lancet.

[ref-19] Sun S, Wu Y, Ma Y, Wang L, Gao Z, Xia C (2016). Impact of degree heterogeneity on attack vulnerability of interdependent networks. Scientific Reports.

[ref-20] Wilcken B (2001). Rare diseases and the assessment of intervention: what sorts of clinical trials can we use?. Journal of Inherited Metabolic Disease.

