# Supplementary material for: In silico study of medical decision-making for rare diseases: heterogeneity of decision-makers in a population improves overall benefit
Source: PeerJ. 2018 Sep 25;6:e5677. doi: 10.7717/peerj.5677 (PMC6161698; doi:10.7717/peerj.5677)
Supplement: Note S1 [file peerj-06-5677-s001.docx]

**Supplementary note 1:**

In the study, decision rules of selecting one out of two new treatments were based on the Beta posterior success rate distribution of each treatment.

**Steps of the proof of Beta conjugate to Bernoulli/Binomial distribution**

Step 1: Bayes’ theorem is known as

$$p\left( \theta| s \right)=\frac{p\left( s | \theta\right)p\left( \theta\right)}{p\left( s \right)}$$

$$=\frac{p(s,\theta)}{\int_{0}^{1} p\left( s,\theta\right)d(\theta)}$$

Where the posterior is proportional to the prior multiplied by likelihood. $p\left( \theta| s \right)$ is posterior distribution, $p\left( s,\theta\right)$is joint distribution, $p\left( \theta\right)$ is prior distribution.$s$ is observed successes, and $\theta$ is the parameter of unknown success rate of each treatment $\left( 0\leq\theta\leq1 \right)$.

Step 2: The binary outcome of each treatment follows a Bernoulli/Binomial likelihood function:

$$p\left( s | \theta\right)={\binom{N}{s}\theta}^{s}\left( 1-\theta\right)^{N-s}$$

$$\frac{N!}{s!\left( N-s \right)!}\theta^{s}\left( 1-\theta\right)^{N-s}$$

$$\frac{\Gamma(N+1)}{\Gamma(s+1)\Gamma(N-s+1)}\theta^{s}{(1-\theta)}^{N-s}$$

where $N$ is the total number of each treatment selected (the treatment is $A$ or $B$), $N-s$ is failures, $s$ is successes, $s=\sum_{i=1}^{N} s_{i}$, and $\theta$ is the parameter of unknown success rate of one treatment $(0\leq\theta\leq1).$

Step 3: In this study we assumed Beta distribution as the prior distribution of each treatment’s success rate:

$$p\left( \theta\right)=\frac{\Gamma(a+b)}{\Gamma(a)\Gamma(b)}\theta^{a-1}{(1-\theta)}^{b-1}$$

$$p\left( \theta\right)\propto\theta^{a-1}{(1-\theta)}^{b-1}$$

where $a$ and $b$ are two parameters of Beta prior distribution. In the study, we assumed a sequential of patients will select one out of two new treatments with no or very limited information, thus the success and failure number both are 0 when the first patient is enrolled in the decision study, then two parameter are $a=b=1$ at the initial prior.

Step 4: Then combine the step 1, step 2 and step 3, the posterior distribution is

$$p\left( \theta| s \right)=p\left( s,\theta\right)=\frac{\Gamma(a+b)\Gamma(N+1)}{\Gamma(a)\Gamma(b)\Gamma(s+1)\Gamma(N-s+1)}\theta^{(a+s-1)}\theta^{(N+b-s-1)}$$

$$p\left( \theta| s \right)\propto\theta^{(a+s-1)}\theta^{(N+b-s-1)}$$

which is also beta distribution with parameters added successes $s$ to $a$ and failures $N-s$ to $b$.

Therefore, the Beta distribution is conjugate distribution to binomial distribution.

In our study, it is reasonable to model the decision rules of selecting one of two treatments based on the Beta posterior distribution, in which the two parameters could be updated by success $s+1$ and failures $f+1$. Also we assume the initial prior parameters $a=b=1$, Beta$(1,1)$ which in fact means that the chance to obtain the success and failure is equal for the first patient.

In the method of this paper, the posterior Beta distribution of success rate for each treatment is written as

$$p_{*}(\theta|n_{*s},n_{*f})=\frac{1}{B(n_{*s}+1, n_{*f}+1)} \theta^{n_{*s}}{(1-\theta)}^{n_{*f}}$$

where $*$indicates $A$ or$B$, $\beta$and $B$ indicate the beta distribution and function, respectively, and $0 \leq\theta\leq1$.
