# Supplementary material for: In silico study of medical decision-making for rare diseases: heterogeneity of decision-makers in a population improves overall benefit
Source: PeerJ. 2018 Sep 25;6:e5677. doi: 10.7717/peerj.5677 (PMC6161698; doi:10.7717/peerj.5677)
Supplement: Note S2 [file peerj-06-5677-s002.docx]

**Supplementary note 2**

**The transition of** $\boldsymbol{2\times2}$ **tables and their probability.**

**
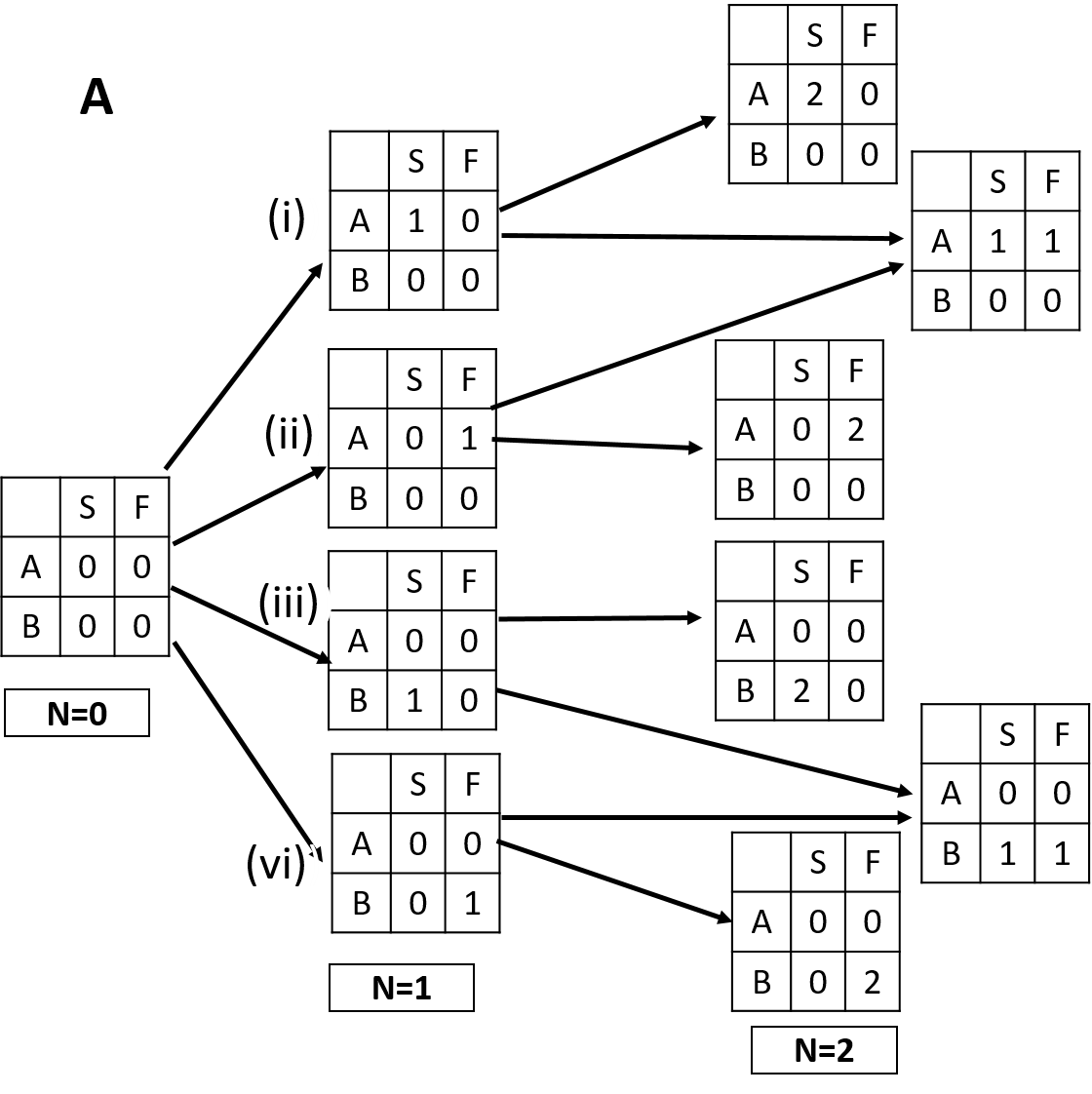
**
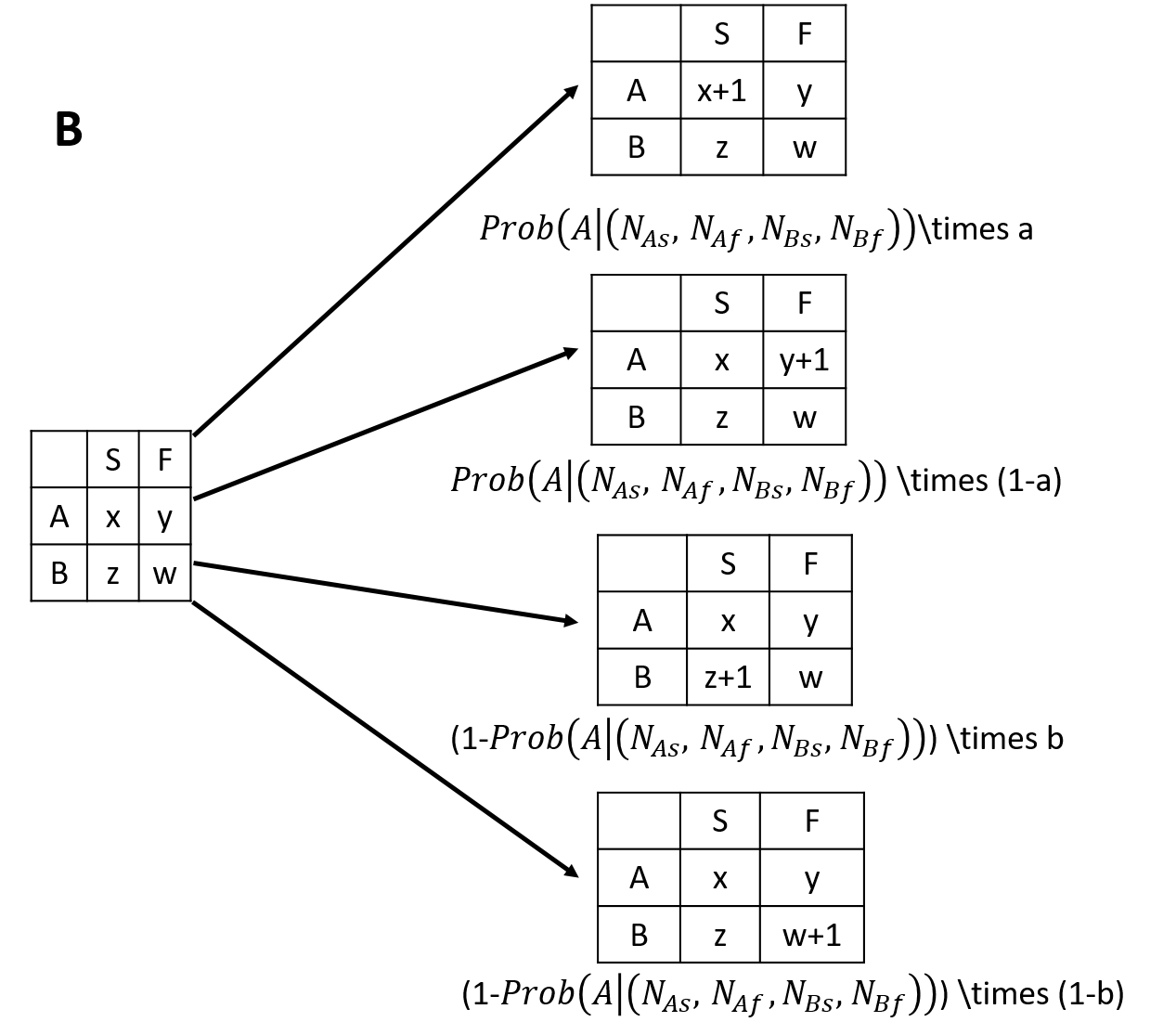


**Legend:** The panel A indicates the transition of $2\times2$ tables. It starts from the left table with four cells all zero due to no outcomes of patient’s selection at the state of $N=0$. When a new patient $(N=1)$ is enrolled, the state should be shifted to right by one step. For each step, there are four possible events when a patient is enrolled; (i) it selects A and turns out to be a success, (ii) it selects A and turns out to be a failure, (iii) it selects B and turns out to be a success and (iv) it selects B and turns out to be a failure. When the second patient entries there are 8 possible events but six types of tables shown as the right side at the state of total patient number $N=2$. Each arrow indicates each possible event and shows connection between the occurred event table and its prior table. Similarly, the number of possible states consisted of $2\times2$ table increases with the increase of the number of patients as shown. The panel B indicates the four possible events and their transition probabilities. When $Prob\left( A | \left( N_{As}, N_{Af}, N_{Bs},N_{Bf} \right) \right)$is explicitly given, the occurrence probability of every $2\times2$ table can be calculated exactly. $Prob\left( A | \left( N_{As}, N_{Af}, N_{Bs},N_{Bf} \right) \right)$is explicitly given for *E.st* population and also for *T.st* population with fixed *w* values. Therefore, the exact probability of all table states is calculable for them. In case of $T.st$ with heterogeneous optimism/pessimism attitudes, $w$ values vary among individuals and $Prob\left( A | \left( N_{As}, N_{Af}, N_{Bs},N_{Bf} \right) \right)$ vary among individuals stochastically, that makes the calculation of exact probability of each tables impossible.
